# Plasma-free anisotropic selective-area etching of β-Ga_2_O_3_ using forming gas under atmospheric pressure

**DOI:** 10.1080/14686996.2024.2378683

**Published:** 2024-07-26

**Authors:** Takayoshi Oshima, Rie Togashi, Yuichi Oshima

**Affiliations:** a Research Center for Electronic and Optical Materials, National Institute for Materials Science, Tsukuba, Japan; b Department of Engineering and Applied Sciences, Sophia University, Chiyoda-ku, Japan

**Keywords:** β-Ga_2_O_3_, forming gas, anisotropic etching, plasma-free process

## Abstract

We demonstrate a facile and safe anisotropic gas etching technique for β-Ga_2_O_3_ under atmospheric pressure using forming gas, a H_2_/N_2_ gas mixture containing 3.96 vol% H_2_. This etching gas, being neither explosive nor toxic, can be safely exhausted into the atmosphere, simplifying the etching system setup. Thermodynamic calculations confirm the viability of gas-phase etching above 676°C without the formation of Ga droplets. Experimental verification was achieved by etching ($\overset{ˉ}{1}$02) β-Ga_2_O_3_ substrates within a temperature range of 700–950°C. Moreover, selective-area etching using this method yielded trenches and fins with vertical and flat sidewalls, defined by (100) facets with the lowest surface energy density, demonstrating significant anisotropic etching capability.

## IMPACT STATEMENT

This paper introduces a safe, plasma-free anisotropic etching technique for β-Ga_2_O_3_ using non-toxic forming gas, achieving high precision and efficiency in semiconductor processing.

## GRAPHICAL ABSTRACT

**Figure:**
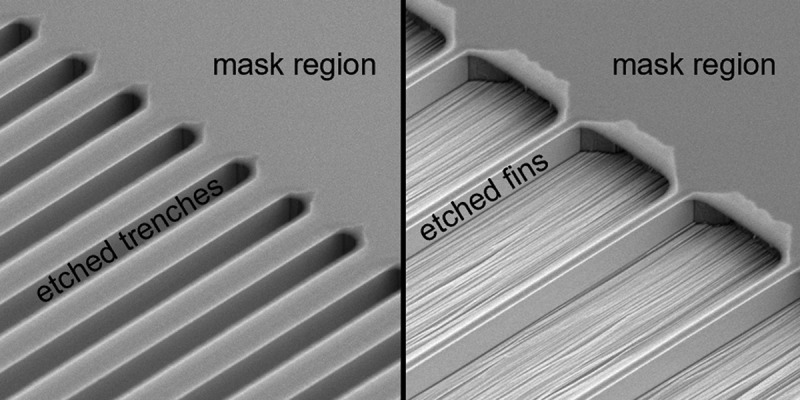

## Introduction

In the past decade, β-Ga_2_O_3_ has emerged as a material of significant interest in power electronic devices owing to its ultrawide bandgap and high breakdown voltage, surpassing those of SiC and GaN [1]. These attributes render β-Ga_2_O_3_ suitable for use in high-power transistors and rectifiers. However, the successful integration of β-Ga_2_O_3_ into semiconductor devices requires precise control of surface characteristics, such as doping concentrations and microstructures. The electron carrier density in β-Ga_2_O_3_ can vary from 10^16^ to 10^20^ cm^−3^ through intentional impurity doping during epitaxy [2] or ion-implantation techniques [3]. Achieving room-temperature p-type conductivity remains challenging because of the deep acceptor levels of potential dopants [4,5] and the expected heavy hole mass owing to the nearly flat dispersion at the upper end of the valance band [6], limiting the ability to enhance the withstand voltage of β-Ga_2_O_3_ power devices while maintaining low on-resistance. For example, mesa-terminated Ni-contact Schottky barrier diodes (SBDs) have shown a maximum practical reverse electric field of 3.4 MV cm^−1^, which is significantly lower than the predicted intrinsic breakdown electric field of 6–8 MV cm^−1^ owing to Schottky barrier tunneling, as described by the field emission model [7]. However, adopting trench metal-oxide-semiconductor (MOS) structures on the surface can mitigate leakage currents in trench SBDs because the current paths are effectively depleted from their sidewalls under reverse bias, thereby enhancing the breakdown voltage [8]. This approach also applies to fin-field-effect transistors (FinFETs), where gate MOS structures between fin sidewalls can enable a normally off operation, and increase breakdown voltage without needing pn homojunctions [9]. Consequently, microfabrication is crucial for improving the performance of β-Ga_2_O_3_-based unipolar devices.

Plasma-based dry etching, a common microfabrication technique, can create the aforementioned trenches and fins [10]. Despite its effectiveness, plasma etching can damage the material surface, or increase defects that function as trap states [11], compromising device performance, as evidenced by the increased on-resistances in trench SBDs [12] and degraded effective channel mobilities and transfer characteristics with large hysteresis in FinFETs [13,14]. Therefore, researchers are increasingly interested in exploring alternative etching methodologies for circumventing plasma-induced damage.

Non-plasma etching is a distinctive method for patterning and shaping the β-Ga_2_O_3_ surface, preserving the surface quality of the material. Wet etching techniques, such as hot phosphoric acid etching [15] and metal-assisted chemical etching (MacEtch) [16,17], can produce microstructures with damage-free sidewalls. However, these sidewalls are positively tapered, rendering them less suitable for device integration. In contrast, plasma-free dry etching methods include Ga-flux etching [18,19], hydrogen environment anisotropic thermal etching (HEATE) [20,21], and HCl gas etching [22–25]. These methods enable the creation of high-aspect-ratio structures with vertical sidewalls devoid of plasma damage, facilitated by directional Ga-beam flux or the manifestation of specific facet planes with low surface energy densities, such as (100), ($\overset{ˉ}{1}$01), {310} facets [26–28]. Nonetheless, these techniques require vacuum equipment and/or gas safety systems, increasing costs and limiting their widespread use.

We overcame these limitations by investigating forming gas as an etching medium under atmospheric pressure, avoiding the need for flammable or toxic gases. Forming gas comprises an inert gas (N_2_ or Ar) mixed with H_2_ and contains a low H_2_ volume of 5% or less, classifying it as nonflammable because of its concentration being close to the lower explosive limit in air (4.0 vol%). This mixture is widely used across various fields for diverse purposes [29–31]. In the context of β-Ga_2_O_3_ device processing, forming-gas treatment at 250°C reduces the interface state density at Al_2_O_3_/β–Ga_2_O_3_ interfaces [32]. However, the patterning of β-Ga_2_O_3_ using forming-gas etching is yet to be reported.

This study demonstrates the effectiveness of selective-area forming-gas etching on β-Ga_2_O_3_ substrates. Thermodynamic calculations were employed to establish the process temperature range. Systematic etching experiments were conducted to explore the etching rate and in-plane anisotropy, which were found to depend on the process temperature. The resulting trenches and fins aligned along specific window directions featured vertical and planar sidewalls, making them suitable for device applications.

## Thermodynamic analysis

**Figure 1.**
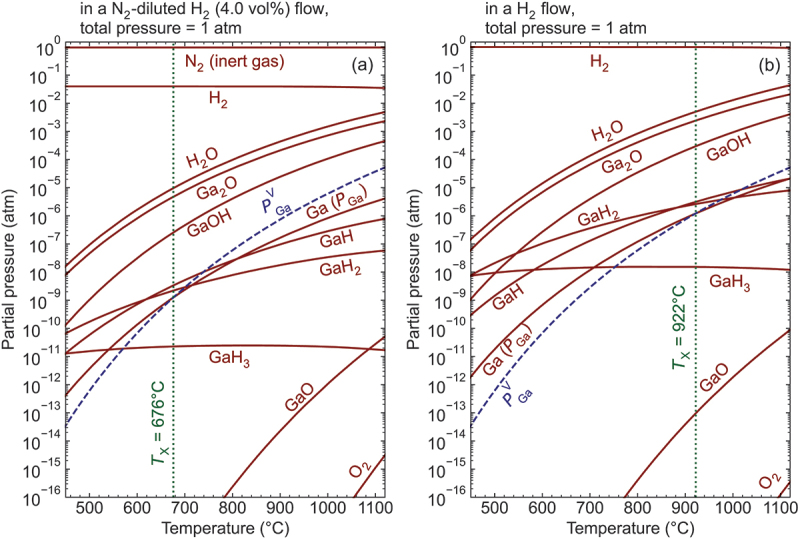
Equilibrium partial pressures of gaseous species above β-Ga_2_O_3_ in (a) N_2_-diluted H_2_ gas flow with 4.0 vol% H_2_ and (b) undiluted H_2_ gas flow under 1 atm as functions of temperature. Red solid lines represent the partial pressures of gaseous species, while the vapor pressure of pure Ga metal ($P_{Ga}^{V}$) is shown with blue dashed lines. The green dotted line *T*_X_ indicates the temperature at which the partial pressure of Ga ($P_{\mathrm{Ga}}$) equals $P_{Ga}^{V}$.

Before the etching experiments, we assessed the thermal and chemical stabilities of β-Ga_2_O_3_ in the presence of H_2_ gas through thermodynamic analysis to determine an appropriate process window. The equilibrium partial pressures of potential gaseous species (Ga_2_O, GaO, Ga, O_2_, H_2_, H_2_O, GaH, GaH_2_, GaH_3_, and GaOH) involved in chemical reactions above the β-Ga_2_O_3_ surface, along with inert N_2_, were calculated following methodologies reported in the literature [33,34]. Figures 1(a,b) depict the pressure curves in N_2_-diluted H_2_ (4.0 vol%) flow and pure H_2_ flow under atmospheric pressure, respectively, as a function of temperature. In both scenarios, the partial pressures of Ga_2_O and H_2_O gases exceed those of other Ga- and O-containing gas species within the examined temperature range, indicating that H_2_ etching predominantly occurs through the formation and desorption of Ga_2_O and H_2_O gases. Focusing on the calculated partial pressure of Ga gas ($P_{\mathrm{Ga}}$) and the literature-reported vapor pressure of pure Ga metal ($P_{Ga}^{V}$) [35], the two values intersect ($P_{\mathrm{Ga}}$ = $P_{Ga}^{V}$) at threshold temperatures (*T*_X_) of 676°C for N_2_-diluted H_2_ and 922°C for pure H_2_. Note that the N_2_ dilution reduces the etching rate due to the lower input H_2_ partial pressure, reducing $P_{\mathrm{Ga}}$ and *T*_X_. *T*_X_ is a pivotal parameter for H_2_ etching, delineating the temperature regions where Ga droplets form ($P_{\mathrm{Ga}}$ > $P_{Ga}^{V}$) below *T*_X_, and where Ga metal is completely evaporated $(P_{\mathrm{Ga}}$ < $P_{Ga}^{V}$) above *T*_X_. Consequently, vapor-phase etching should be conducted above *T*_X_, with N_2_ dilution serving to lower *T*_X_. Based on these thermodynamic considerations, we established the process temperature range between 700°C and 950°C (above *T*_X_) to prevent Ga droplet formation.

## Experimental methods

This study employed β-Ga_2_O_3_ substrates with a ($\overset{ˉ}{1}$02) orientation, supplied by Novel Crystal Technology, Inc. The ($\overset{ˉ}{1}$02) plane is parallel to the [010] axis and perpendicular to the (100) plane, which is known for its lowest surface energy density [26]. This orientation coincides with the primary dislocation and void/nanopipe directions within the crystal [36–38], making it particularly suitable for vertical anisotropic etching. This avoids undesired crystal defects on the surface, favoring vertical trench and fin device applications [25]. For detailed methodologies and findings from our previous experiments on ($\overset{ˉ}{1}$02) substrates, including HCl gas etching, readers are referred to our published studies [25,39,40].

The experimental setup began with the preparation of a patterned SiO_2_ mask on Sn-doped ($\overset{ˉ}{1}$02) β-Ga_2_O_3_ substrates with an effective donor concentration of ~4.9 × 10^18^ cm^−3^. A 0.1-μm-thick SiO_2_ layer was deposited onto the substrate surface using high-throughput plasma-assisted chemical vapor deposition. Although the plasma deposition process might damage the β-Ga_2_O_3_ surface, this damage should be independent of the forming-gas etching characteristics, which are the focus of this study. For practical applications, SiO_2_ layers should be deposited using non-plasma techniques, such as atomic layer deposition using O_3_ as an oxidant. The deposited SiO_2_ layer was patterned through standard laser lithography and buffered HF etching to create masks with various window shapes. These included square, circular, radial-line windows, and two types of striped windows (Pattern T and Pattern F), designed to explore different etched structures. The dimensions for the square windows were 100 × 100 μm^2^, the circular windows had a diameter of 1.5 μm, and the radial-line windows were 1.2 μm wide. The striped windows, intended for trench and fin fabrication, had window/mask widths of 1.2/1.8 μm for Pattern T and 5.5/0.7 μm for Pattern F, respectively. See Figure S1 in the supplementary file for diagrams of these window shapes.

**Figure 2.**
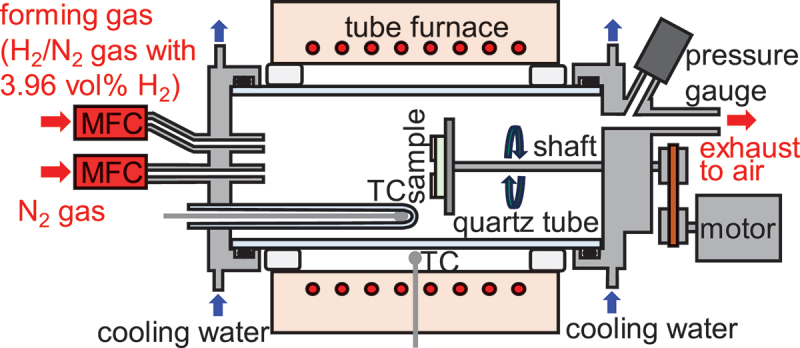
Schematic of the forming-gas etching system utilized in this research.

Etching was performed in a horizontal quartz furnace equipped with a gas flow system, as illustrated in Figure 2. The substrate was heated to target temperatures of 700°C, 750°C, 800°C, 875°C, and 950°C under a 1.000 slm flow of inert N_2_ gas. The temperature was monitored by a thermocouple (TC) inside the quartz tube, calibrated against a reference thermocouple placed at the sample location prior to experimentation. The etching commenced upon switching the gas supply from pure N_2_ to forming gas – a mixture of H_2_ and N_2_ with 3.96 vol% H_2_—while maintaining a 1.000 slm flow rate. The exhaust was vented directly into the atmosphere. The process is concluded by reverting the gas supply to pure N_2_ and allowing the furnace to cool to ambient temperature. This entire procedure was conducted at atmospheric pressure (~1 atm), distinguishing it from other vapor-phase etching methods by obviating the need for vacuum systems and gas safety mechanisms, thereby reducing costs.

Post-etching analyses involved measuring the depth within the square windows (100 × 100 μm^2^) using a stylus profiler and atomic force microscopy to determine the etching rate. The etched structures were then examined with scanning electron microscopy (SEM), setting the acceleration voltage to 10 kV to clearly delineate the etched hole edges without mask removal [22]. Cross-sectional views of the etched structures were revealed by focused ion beam milling and further observed via SEM.

## Experimental results and discussion

### Etching rate of the ($\overset{ˉ}{1}$02) surface

**Figure 3.**
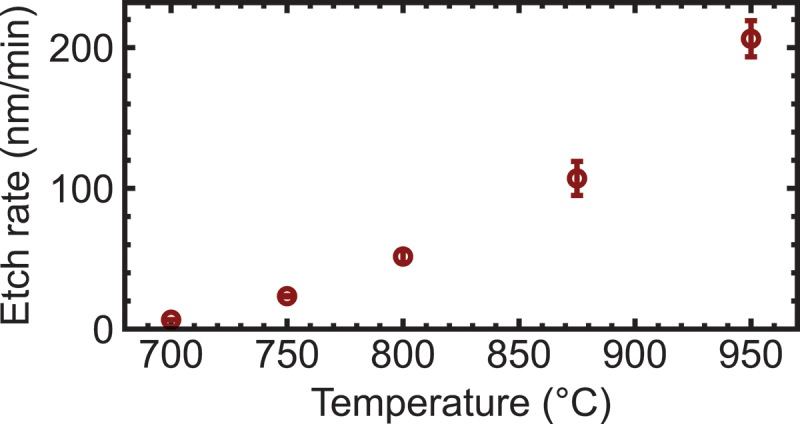
Etching rate of ($\overset{ˉ}{1}$02) β-Ga_2_O_3_ plotted against process temperature.

Initially, we measured the etching rate of the ($\overset{ˉ}{1}$02) surface across a temperature range of 700°C–950°C. Notably, no Ga droplets were detected on the etched surfaces of any samples, consistent with predictions from thermodynamic analyses. The etching rate showed a pronounced increase with elevated process temperatures. They were 6.7 ± 0.1 nm/min at 700°C, 23.4 ± 0.4 nm/min at 750°C, 51.6 ± 4.2 nm/min at 800°C, 107.1 ± 12.0 nm/min at 875°C, and 206.3 ± 12.8 nm/min at 950°C (Figure 3). This result indicates a significant enhancement of the etching reaction by thermal energy within this temperature range. To elucidate the trends associated with the process temperature, we compare structures etched at 750°C for 60 min and at 950°C for 5 min in the subsequent sections.

### In-plane etching characteristics

**Figure 4.**
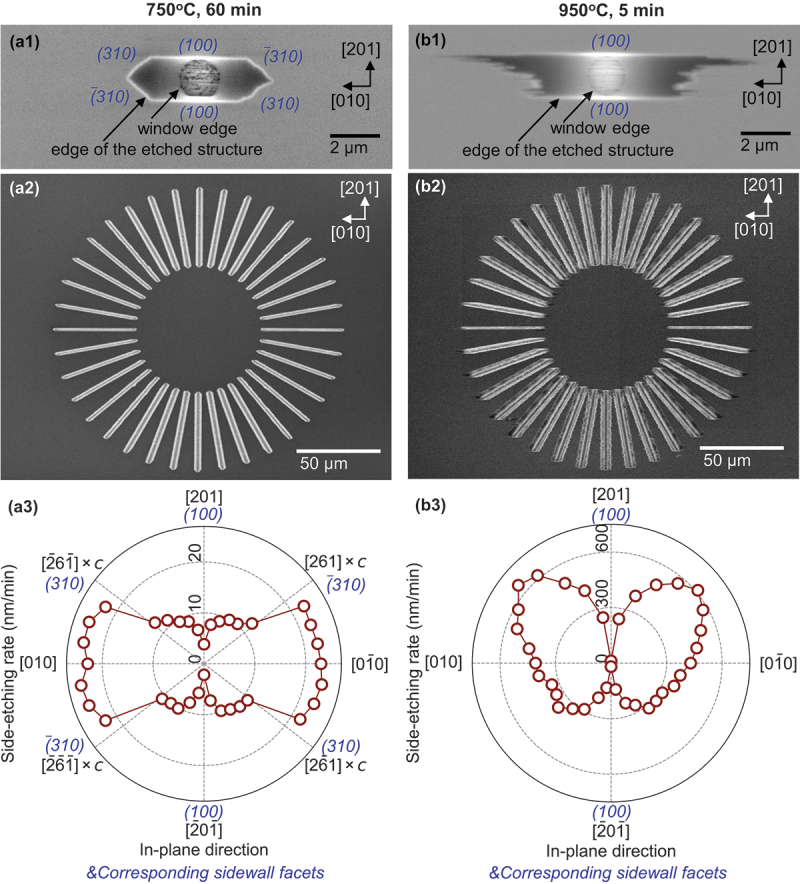
SEM images of etched structures on ($\overset{ˉ}{1}$02) β-Ga_2_O_3_ substrate: (a1) and (a2) show top views under a circular window and radial-line windows, respectively, processed at 750°C for 60 min. (a3) presents a polar plot of the side-etching rate derived from (a2). Images (b1)–(b3) correspond to samples processed at 950°C for 5 min. Sidewall facets are denoted by Miller indices in blue.

We further explored the in-plane etching behavior by examining the side-etched shapes from a surface-normal perspective. The etched shapes reflected the asymmetry of the β-Ga_2_O_3_ crystal structure, which has two symmetry elements: two-fold rotation symmetry around the [010] axis and mirror symmetry across the (010) plane. Figure 4 summarizes the etched structures beneath circular and radial-line windows at process temperatures of 750°C and 950°C. In all the images, side-etched shapes are symmetrical in the [010] and [0$\overset{ˉ}{1}$0] directions, reflecting the mirror symmetry across the (010) plane.

At 750°C, the etched structures reflected the in-plane anisotropy of the crystal. Figure 4(a1) shows a top-view SEM image of an etched hole beneath a circular window, where the contour of the etched shape forms a hexagon elongated along the [010] direction. This shape points to the emergence of (100)- and {310}-faceted sidewalls, similar to findings from our previous HCl gas etching study on ($\overset{ˉ}{1}$02) substrates [25]. The (100) plane has the lowest surface energy density [26], while {310} planes, being oxygen-close-packing planes, are expected to have relatively low surface energy densities [28]. The detailed in-plane dependence of the side-etching rate, derived from the SEM image of radial-line windows (Figure 4(a2)), is represented in polar coordinates (Figure 4(a3)). The polar plot reveals deep and shallow dips corresponding to (100) and {310} sidewalls, respectively, aligning with the elongated hexagonal shape observed under the circular window Figure 4(a1). Conversely, broad peaks around the <010> direction indicate an increased side-etching rate of the (010) plane.

At 950°C, except for the (100) facets, the sidewalls of the etched structures appeared roughened. Figure 4(b1) illustrates the etched depression under a circular window, where the contour in the <201> directions appears linear, delineated by (100) facets, while significant roughness is evident in the <010> directions. Moreover, side-etching on the [201] side surpassed that on the [$\overset{ˉ}{2}$0$\overset{ˉ}{1}$] side. This structure suggests the absence of {310} sidewalls due to enhanced side-etching on the (010) plane at the elevated process temperature. Note that the asymmetry of the side-etching structures between the [$\overset{ˉ}{2}$0$\overset{ˉ}{1}$] and [201] direction sides is attributed to the two-fold rotation symmetry, where the crystal lattices on the [$\overset{ˉ}{2}$0$\overset{ˉ}{1}$] and [201] direction sides are rotated 180° around the [010] axis with respect to each other. Given that the etching beneath the mask proceeded not only in a lateral direction but also in a downward direction, it is likely that different crystal planes were etched between the [$\overset{ˉ}{2}$0$\overset{ˉ}{1}$] and [201] direction sides owing to the asymmetric lattice structure, resulting in a marked difference in the side-etching structures. Figures 4(b2) and 4(b3) display etched trenches under radial-line windows and a polar plot of their side-etching rates, respectively. Trenches along <010> directions showed minimal side-etching rates due to (100)-faceted sidewalls, while side-etching rates increased for trenches deviating from <010> directions. Additionally, trench sidewalls near the [201] direction exhibited a zigzag pattern. A distinct butterfly-wing-like pattern in the polar plot aligns well with the etched structure observed under the circular window. Notably, the polar plot at 750°C did not show a significant difference in side-etching rates between the [201] and [$\overset{ˉ}{2}$0$\overset{ˉ}{1}$] directions owing to the existence of relatively stable {310} sidewalls. These results indicate that higher process temperatures alter the etching behavior, including in-plane etching anisotropy, while (100) facets remain unaffected.

### Etched trenches and fins

**Figure 5.**
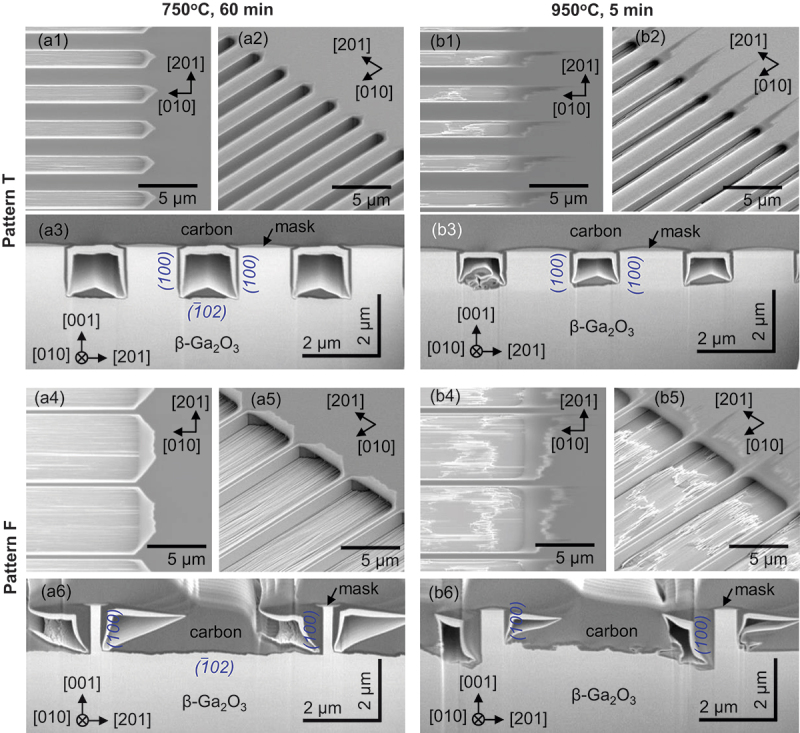
SEM images of etched structures beneath pattern T (a1)–(a3) and pattern F (a4)–(a6) striped windows along [010] direction at 750°C for 60 min. Images (a1) and (a4) show top views, (a2) and (a5) are tilted surface views, and (a3) and (a6) depict tilted cross-sectional views. Images (b1)–(b5) represent samples processed at 950°C for 5 min. Note the difference in vertical and horizontal scales in the cross-sectional views due to the tilted perspective.

We further examined the structures of trenches and fins with (100)-faceted sidewalls to evaluate the potential of forming-gas etching for semiconductor processing. Figure 5 illustrates the etched structures beneath striped windows (Patterns T and F) at process temperatures of 750°C and 950°C.

The trenches and fins were successfully created in accordance with the design of the windows and masks. Surface SEM images showed minimal side-etching perpendicular to the [010] direction windows, less than 0.2 μm, regardless of the process temperature and window patterns, attributed to the formation of (100)-faceted sidewalls Figures 5(a1), 5(a4), 5(b1), and 5(b4)). This precision in patterning trenches and fins, with their widths defined accurately by the windows and masks, is advantageous for semiconductor device fabrication. Furthermore, the (100) sidewalls were observed to be perfectly flat (Figures 5(a2), 5(a5), 5(b2), and 5(b5)), a characteristic beneficial for device applications due to the implied low surface state density on these sidewalls. However, the bottom surface of the etched areas showed relative roughness, which increased with the process temperature (Figures 5(a4),5(a5), 5(b4), and 5(b5)). Such surface roughness is consistent with observations from HCl gas etching on ($\overset{ˉ}{1}$02) substrates [25], suggesting that roughening is a typical result of gas etching on the ($\overset{ˉ}{1}$02) plane. The observed roughening of the ($\overset{ˉ}{1}$02) bottom surfaces relative to the (100) sidewall surfaces at elevated temperatures can be attributed to the difference in the roughening transition temperatures associated with the free energies of steps on the respective planes. On the ($\overset{ˉ}{1}$02) bottom plane, the step sidewalls are predominantly formed with (100) facets (Figure 5). Since the (100) facet has the lowest surface energy density, the free energies of the steps with (100) sidewall facets on the ($\overset{ˉ}{1}$02) bottom plane are relatively low, decreasing the roughening transition temperature. Conversely, on the (100) sidewall plane, the free energies of steps must be larger because the step sidewalls comprise facets other than the (100) facets, leading to a higher roughening transition temperature than that on the ($\overset{ˉ}{1}$02) plane.

Cross-sectional analysis highlighted the contours of these trenches and fins. The (100) sidewalls on both sides were perfectly vertical (Figures 5(a3), 5(a6), 5(b3), and 5(b6)), demonstrating effective anisotropic etching. Notably, the profiles from the 750°C etching, with smoother bottoms, were nearly ideal. The asymmetry of the etched trenches and fins between the [$\overset{ˉ}{2}$0$\overset{ˉ}{1}$] and [201] directional sides was also observed, particularly at the 950°C etching. This result is consistent with the in-plane etching characteristics discussed in the previous section. The aspect ratio of vertical anisotropy, defined as the height of the (100) sidewall divided by the side-etched length, varied from 7.0 to 8.4 at 750°C and from 13 to 30 at 950°C. These values surpass the aspect ratios of 2.7 to 6.7 reported for HCl gas etching on ($\overset{ˉ}{1}$02) substrates at 1038°C [25]. The capability of achieving vertical etching with smooth sidewall profiles highlights the potential of forming-gas etching as a viable plasma-free anisotropic dry etching technique.

## Summary

We introduced a novel plasma-free anisotropic etching technique for ($\overset{ˉ}{1}$02) β-Ga_2_O_3_ substrates utilizing forming gas (a N_2_/H_2_ mixture containing 3.96% H_2_) at atmospheric pressure. Vapor-phase etching without the formation of Ga droplets was achieved at process temperatures of 700°C and above, aligning with thermodynamic predictions. The etched structures demonstrated anisotropic features reflective of the β-Ga_2_O_3_ crystal structure, with side-etching minimized along the <010> direction due to the formation of (100) faceted sidewalls. This facilitated precise patterning and the creation of high-aspect-ratio trenches and fins with vertical and smooth sidewalls, devoid of plasma-induced damage. The use of forming gas simplifies the etching process, leveraging the nontoxic and nonflammable properties of the gas. This method is expected to gain traction within the β-Ga_2_O_3_ research community.

## Supplementary Material

Supplemental Material

## References

[1] Green AJ, Speck J, Xing G, et al. β-gallium oxide power electronics. APL Mater. 2022;10(2):029201. doi: 10.1063/5.0060327

[2] Feng Z, Anhar Uddin Bhuiyan AFM, Karim MR, et al. MOCVD homoepitaxy of Si-doped (010) β-Ga2O3 thin films with superior transport properties. Appl Phys Lett. 2019;114(25):250601. doi: 10.1063/1.5109678

[3] Sasaki K, Higashiwaki M, Kuramata A, et al. Si-ion implantation doping in β-Ga2O3 and its application to fabrication of low-resistance ohmic contacts. Appl Phys Express. 2013;6(8):086502. doi: 10.7567/APEX.6.086502

[4] Peelaers H, Lyons JL, Varley JB, et al. Deep acceptors and their diffusion in Ga2O3. APL Mater. 2019;7(2):22519. doi: 10.1063/1.5063807

[5] Tadjer MJ, Lyons JL, Nepal N, et al. Theory and characterization of doping and defects in β-Ga2O3. ECS J Solid State Sci Technol. 2019;8(7):Q3187. doi: 10.1149/2.0341907jss

[6] Mohamed M, Unger I, Janowitz C, et al. The surface band structure of β-Ga2O3. J Phys: Conf Ser. 2011;286:012027. doi: 10.1088/1742-6596/286/1/012027

[7] Li W, Saraswat D, Long Y, et al. Near-ideal reverse leakage current and practical maximum electric field in β-Ga2O3 schottky barrier diodes. Appl Phys Lett. 2020;116(19):192101. doi: 10.1063/5.0007715

[8] Otsuka F, Miyamoto H, Takatsuka A, et al. Large-size (1.7 × 1.7 mm2) β-Ga2O3 field-plated trench MOS-type schottky barrier diodes with 1.2 kV breakdown voltage and 10^9 high on/off current ratio. Appl Phys Express. 2022;15(1):016501. doi: 10.35848/1882-0786/ac4080

[9] Li W, Nomoto K, Hu Z, et al. Single and multi-fin normally-off Ga2O3 vertical transistors with a breakdown voltage over 2.6 kV. In: 2019 IEEE International Electron Devices Meeting (IEDM). San Francisco (USA): IEEE. p.12.4.1–.12.4.4.

[10] Hogan JE, Kaun SW, Ahmadi E, et al. Chlorine-based dry etching of β-Ga2O3. Semicond Sci Technol. 2016;31(6):065006. doi: 10.1088/0268-1242/31/6/065006

[11] Wang Z, Yu X, Gong H, et al. Identification and suppression of majority surface states in the dry-etched β-Ga2O3. J Phys Chem Lett. 2022;13(30):7094. doi: 10.1021/acs.jpclett.2c02167 35900195

[12] Li W, Nomoto K, Hu Z, et al. ON-Resistance of Ga2O3 trench-MOS schottky barrier diodes: role of sidewall interface trapping. IEEE Trans Electron Devices. 2021;68(5):2420. doi: 10.1109/TED.2021.3067856

[13] Hu Z, Nomoto K, Li W, et al. Breakdown mechanism in 1 kA/cm2 and 960 V E-mode β-Ga2O3 vertical transistors. Appl Phys Lett. 2018;113(12):122103. doi: 10.1063/1.5038105

[14] Chabak KD, Moser N, Green AJ, et al. Enhancement-mode Ga2O3 wrap-gate fin field-effect transistors on native (100) β -Ga2O3 substrate with high breakdown voltage. Appl Phys Lett. 2016;109(21):213501. doi: 10.1063/1.4967931

[15] Oshima T, Okuno T, Arai N, et al. Wet etching of β-Ga2O3 substrates. Jpn J Appl Phys. 2009;48(4R):040208. doi: 10.1143/JJAP.48.040208

[16] Huang H, Kim M, Zhan X, et al. High aspect ratio β-Ga2O3 fin arrays with low-interface charge density by inverse metal-assisted chemical etching. ACS Nano. 2019;13(8):8784. doi: 10.1021/acsnano.9b01709 31244033

[17] Huang H-C, Ren Z, AnharUddin Bhuiyan AFM, et al. β-Ga2O3 FinFETs with ultra-low hysteresis by plasma-free metal-assisted chemical etching. Appl Phys Lett. 2022;121(5):052102. doi: 10.1063/5.0096490

[18] Kalarickal NK, Fiedler A, Dhara S, et al. Planar and three-dimensional damage-free etching of β-Ga2O3 using atomic gallium flux. Appl Phys Lett. 2021;119(12):123503. doi: 10.1063/5.0057203

[19] Dhara S, Kalarickal NK, Dheenan A, et al. β-Ga2O3 trench schottky diodes by low-damage Ga-atomic beam etching. Appl Phys Lett. 2023;123(2):023503. doi: 10.1063/5.0151808

[20] Ooe Y, Kawasaki Y, Moriya Y, et al. Hydrogen environment anisotropic thermal etching of (010) β-Ga2O3 and fabrication of high-aspect Ga2O3 nanowall structures. In: The 3rd International Workshop on Gallium Oxide and Related Materials, DEV 37. Columbus (USA); 2019.

[21] Yamazaki Y, Tomoaki M, Takeki A, et al. Fabrication of high aspect DBR structures for optical integrated devices by hydrogen environment anisotropic thermal etching of β-Ga2O3. In: The 4th International Workshop on Gallium Oxide and Related Materials, Proc. Nagao (Japan); 2022. p. 1–8.

[22] Oshima T, Oshima Y. Plasma-free dry etching of (001) β-Ga2O3 substrates by HCl gas. Appl Phys Lett. 2023;122(16):162102. doi: 10.1063/5.0138736

[23] Oshima T, Oshima Y. Anisotropic non-plasma HCl gas etching of a (010) β-Ga2O3 substrate. Appl Phys Express. 2023;16(6):066501. doi: 10.35848/1882-0786/acdbb7

[24] Oshima Y, Oshima T. Effect of the temperature and HCl partial pressure on selective-area gas etching of (001) β-Ga2O3. Jpn J Appl Phys. 2023;62(8):080901. doi: 10.35848/1347-4065/acee3b

[25] Oshima T, Oshima Y. Using selective-area growth and selective-area etching on (−102) β-Ga2O3 substrates to fabricate plasma-damage-free vertical fins and trenches. Appl Phys Lett. 2024;124(4):042110. doi: 10.1063/5.0186319

[26] Mu S, Wang M, Peelaers H, et al. First-principles surface energies for monoclinic Ga2O3 and Al2O3 and consequences for cracking of (AlxGa1−x)2o3. APL Mater. 2020;8(9):091105. doi: 10.1063/5.0019915

[27] Schewski R, Lion K, Fiedler A, et al. Step-flow growth in homoepitaxy of β-Ga2O3 (100)—the influence of the miscut direction and faceting. APL Mater. 2019;7(2):022515. doi: 10.1063/1.5054943

[28] Yamaguchi H, Kuramata A, Masui T. Slip system analysis and X-ray topographic study on β-Ga2O3. Superlattices Microstruct. 2016;99:99. doi: 10.1016/j.spmi.2016.04.030

[29] Sana P, Rohatgi A, Kalejs JP, et al. Gettering and hydrogen passivation of edge-defined film-fed grown multicrystalline silicon solar cells by Al diffusion and forming gas anneal. Appl Phys Lett. 1994;64(1):97. doi: 10.1063/1.110880

[30] Onishi K, Kang CS, Choi R, et al. Improvement of surface carrier mobility of HfO2 MOSFETs by high-temperature forming gas annealing. IEEE Trans Electron Devices. 2003;50(2):384. doi: 10.1109/TED.2002.807447

[31] Hwang J, Goh Y, Jeon S. Effect of forming gas high-pressure annealing on metal-ferroelectric-semiconductor hafnia ferroelectric tunnel junction. IEEE Electron Device Lett. 2020;41(8):1193. doi: 10.1109/LED.2020.3001639

[32] Islam AE, Zhang C, DeLello K, et al. Defect engineering at the Al2O3/(010) β-Ga2O3 interface via surface treatments and forming gas post-deposition anneals. IEEE Trans Electron Devices. 2022;69(10):5656. doi: 10.1109/TED.2022.3200643

[33] Togashi R, Nomura K, Eguchi C, et al. Thermal stability of β-Ga2O3 in mixed flows of H2 and N2. Jpn J Appl Phys. 2015;54(4):041102. doi: 10.7567/JJAP.54.041102

[34] Togashi R, Kisanuki Y, Goto K, et al. Thermal and chemical stabilities of group-III sesquioxides in a flow of either N2 or H2. Jpn J Appl Phys. 2016;55(12):1202BE. doi: 10.7567/JJAP.55.1202BE

[35] Knacke O, Kubaschewski O, Hesselmann K. Thermochemical properties of inorganic substances. 2nd ed. Berlin (Germany): Springer-Verlag; 1991.

[36] Yao Y, Tsusaka Y, Sasaki K, et al. Large-area total-thickness imaging and burgers vector analysis of dislocations in β-Ga2O3 using bright-field x-ray topography based on anomalous transmission. Appl Phys Lett. 2022;121(1):012105. doi: 10.1063/5.0098942

[37] Oshima T, Hashiguchi A, Moribayashi T, et al. Electrical properties of schottky barrier diodes fabricated on (001) β-Ga2O3 substrates with crystal defects. Jpn J Appl Phys. 2017;56(8):086501. doi: 10.7567/JJAP.56.086501

[38] Nishikawa T, Goto K, Murakami H, et al. Observation of nanopipes in edge-defined film-fed grown β-Ga2O3 substrate and their effect on homoepitaxial surface hillocks. Jpn J Appl Phys. 2023;62(SF): SF1015. doi: 10.35848/1347-4065/acc18e

[39] Oshima Y, Oshima T. Homoepitaxial growth of (-102) β-Ga2O3 by halide vapor phase epitaxy. Semicond Sci Technol. 2023;38(10):105003. doi: 10.1088/1361-6641/acf241

[40] Oshima T, Nakagomi S. Epitaxial relationship of NiO on (-102) β-Ga2O3. Jpn J Appl Phys. 2023;62(12):128001. doi: 10.35848/1347-4065/ad0ac9

