# Supplementary material for: Plasma-free anisotropic selective-area etching of β-Ga2O3 using forming gas under atmospheric pressure
Source: Sci Technol Adv Mater. 2024 Jul 26;25(1):2378683. doi: 10.1080/14686996.2024.2378683 (PMC11288204; doi:10.1080/14686996.2024.2378683)
Supplement: Supplemental Material [file TSTA_A_2378683_SM7557.pdf]

Supplementary file

Plasma-free anisotropic selective-area etching of β-Ga_2O_3 using forming gas under atmospheric pressure

Takayoshi Oshima[a*], Rie Togashi[b], and Yuichi Oshima[a]

[a]*Research Center for Electronic and Optical Materials, National Institute for Materials Science, Tsukuba, Japan*

[b]*Department of Engineering and Applied Sciences, Sophia University, Chiyoda-ku, Japan*

CONTACT

Takayoshi Oshima

OSHIMA.Takayoshi@nims.go.jp

Supplementary file

1. Window designs

Square, circular, radial-line, striped window patterns utilized in the experiments are shown in Figure S1 for easy understanding.

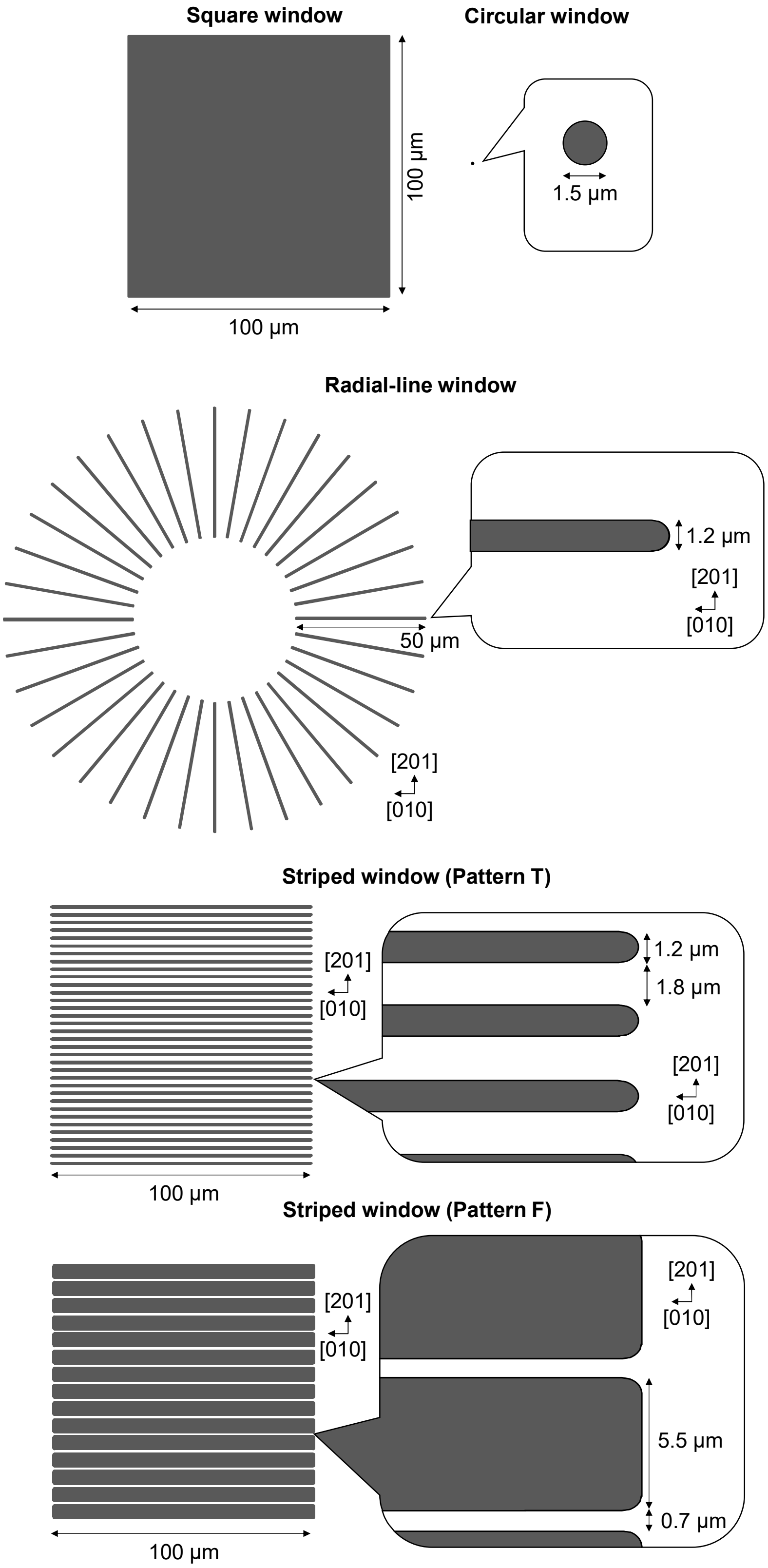

Figure S1. Designs of the window patterns used in the etching experiments. For further details, please refer to the original paper. Note that the ends of the line patterns are curved due to the finite size of the laser spot.
